# How immersive virtual reality can become a key tool to advance research and psychotherapy of eating and weight disorders

**DOI:** 10.3389/fpsyt.2022.1011620

**Published:** 2022-11-03

**Authors:** Simone C. Behrens, Stephan Streuber, Anouk Keizer, Katrin E. Giel

**Affiliations:** ^1^Department of Psychosomatic Medicine and Psychotherapy, University Hospital Tübingen, Tübingen, Germany; ^2^Max Planck Institute for Intelligent Systems, Tübingen, Germany; ^3^Centre of Excellence for Eating Disorders (KOMET), Tübingen, Germany; ^4^Department of Electrical Engineering and Computer Science, Coburg University of Applied Sciences and Arts, Coburg, Germany; ^5^Department of Experimental Psychology, University of Utrecht, Utrecht, Netherlands

**Keywords:** virtual reality, psychotherapy, eating and weight disorders, user-centered design, exposure therapy

## Abstract

Immersive virtual reality technology (VR) still waits for its wide dissemination in research and psychotherapy of eating and weight disorders. Given the comparably high efforts in producing a VR setup, we outline that the technology’s breakthrough needs tailored exploitation of specific features of VR and user-centered design of setups. In this paper, we introduce VR hardware and review the specific properties of immersive VR versus real-world setups providing examples how they improved existing setups. We then summarize current approaches to make VR a tool for psychotherapy of eating and weight disorders and introduce user-centered design of VR environments as a solution to support their further development. Overall, we argue that exploitation of the specific properties of VR can substantially improve existing approaches for research and therapy of eating and weight disorders. To produce more than pilot setups, iterative development of VR setups within a user-centered design approach is needed.

## Introduction

For years, immersive virtual reality technology (VR) has been expected to change psychotherapy (1), but although its use is increasing (2), widespread use of the technology for research and therapy of eating and weight disorders is still a long way off. VR provides a computer-generated 3-dimensional digital environment with which a user can naturally interact. For example, when placed in a scenery, the user does not need to press buttons to see the scenery to his/her left, but it is sufficient to look to the left. However, the impressive experience of VR is offset by high costs for the development of the setups and many promising approaches currently get stuck in their pilot stage. In this perspective paper, we argue that VR will only become prevalent in eating disorder research and treatment if benefits of the technology are exploited more systematically and studies are planned within an iterative process. To this end, we outline the specific properties of VR and provide examples how they can be harnessed for improving research setups and establishing enhanced psychotherapy for eating and weight disorders. To account for the complexity of VR setups, we recommend a user-centered design approach for their development and outline how this can be implemented.

## Hardware for virtual reality

The current state-of-the-art hardware for VR uses head-mounted displays as output devices that immerse the user in a virtual world. Head-mounted devices typically present the user with stereoscopic images, whose pixels are updated according to actual head movements and virtual motion. Anthes and Garcia-Hernandez (3) distinguish two main categories of head-mounted devices: Wired and mobile devices. Wired head-mounted displays typically get their signal from a powerful state-of-the-art gaming personal computer (PC) that is connected *via* a cable. Mobile devices are standalone systems that work without an additional PC. Many mobile devices are based on smartphone technology, which is either integrated into an ergonomic case or indeed a smartphone which is then plugged into a simple frame made of plastic or cardboard. While mobile VR experience is typically inferior to wired devices in terms of computational power and visual realism, a clear advantage of mobile devices is their lower cost and ease of use, since they don’t require a sophisticated setup.

Design of VR environments is significantly intertwined with hardware abilities and constraints. Hardware properties such as resolution, field of view, or weight or tracking capabilities define large parts of the user experience, such as presence, user comfort, risk for cybersickness and opportunities to implement natural interaction. At the same time, high quality VR-experiences often require more complex hardware and more sophisticated setups. The hardware market for VR is highly dynamic, which implies that a thorough analysis of existing and announced devices is crucial to ensure a good match between project requirements and hardware specifications. Websites such as https://vr-compare.com summarize hardware specifications and accessories. Relevant aspects for comparison, which also determine quality of VR experience and user opportunities within VR may be:

• Ergonomics: Headsets differ with respect to form, weight and size. These factors effect user experience and usability. Weight and weight distribution of the headset can lead affect fatigue, strain and discomfort. Display type, configuration and optics can lead to different levels of eyestrain and discomfort. Those factors might negatively affect cognition and attentional process.
• Optics: Some headsets allow for adjustment of interpapillary distance, which may reduce the perception of double images. The higher the range, the more suitable it is for very small and big heads. Adjustable diopters allow to compensate myopia and hyperopia directly on the headset.
• Field of View: A human eye has a horizontal field of view of about 135 degrees (combined 200 degrees) and a vertical field of view of over 180 degrees. Head-mounted displays typically reach considerably less, resulting in the impression to look through a box. A high field of view improves the display quality considerably, although the finally reached field of view may vary between persons. A large field of view increases presence and allows for the perception of visual information about the own virtual body, which might be relevant for eating disorder studies.
• Display: Display type and resolution determine how sharp and colorful the user sees the virtual environment. Generally, higher resolution leads to sharper images. Liquid crystal displays (LCD) produce sharper edges yet less vivid colors compared to (Active Matrix) Organic Light Emitting Diode [(AM)OLED] displays. Some devices support foveated rendering, which reduces resolution in the periphery and thereby computational resources based on eye-tracking.
• Tracking: Older mobile devices track their position only based on rotational tracking through gyroscopes. This allows for natural view, but not for moving around the virtual world physically or for naturally interaction with it. To enable natural interaction, the headset and additional equipment needs to be tracked with 6 degrees of freedom (3 positional, 3 rotational). Tracking is usually implemented using inertia measurement units (IMUs) and/or optical tracking that constantly provide spatial reference. Optical tracking can be implemented through additional external cameras or lighthouses (“outside-in tracking”), or, as in some more recent devices, through cameras or LiDAR built into the headsets (“inside-out tracking”) that relate the rotation and acceleration data to static features of the room. While inside-out tracking maximizes ease of use, outside-in solutions still provide the best accuracy and latency, which reduces the risk for cybersickness and increases data quality of recorded motion. A further relevant aspect is which additional equipment can be tracked and whether or not it needs additional accessories, e.g., position of controllers, eye-movements, hands or other body parts.
• Additional Accessories: A number of additional accessories exist which can be relevant to certain applications. Examples are devices for haptic feedback on the hand or back, omnidirectional treadmills, additional trackers that can be attached to body parts or prescription lens sets. Since not all devices are compatible with all accessories, the need for specific accessories can significantly limit the choice of devices. Note that some headsets can also be used for augmented reality applications.

## Specific properties of virtual reality and their use to research and therapy

**FIGURE 1:**
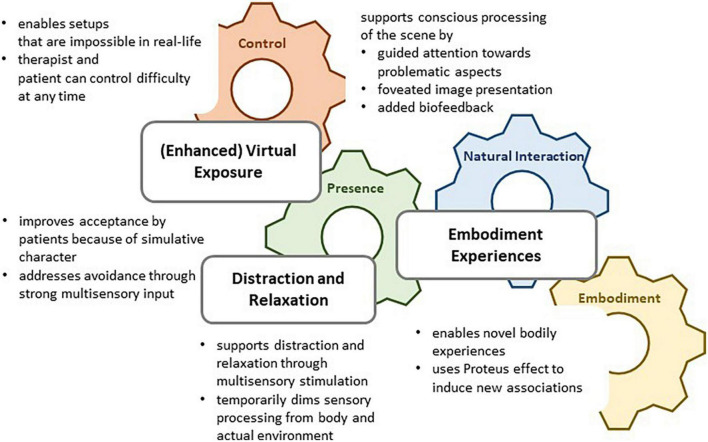
Virtual reality (VR) as a tool for psychotherapy.

Historically, a dedicated aim of VR development is to create a second, illusory reality by replacing multisensory input from the environment through information on the virtual environment (4). Since this replacement is typically incomplete, virtual realities bear specific features that distinguish them from “normal” reality. Here, we emphasize that exactly these differences between virtual and normal reality can be very useful to research and treatment of eating disorders (cf. Figure 1). Specifically, we see four relevant properties of VR that can be harnessed for created innovative approaches in research and therapy: (1) Control, (2) Natural interaction and surveillance, (3) Presence, and (4) Embodiment.

### Control

Virtual environments enable complex situations which are still standardized in what a user can experience and do. This makes them highly relevant for the investigation of eating behavior, which is regulated by a variety of interacting factors. VR also provided extended opportunities for standardizing complex experimental procedures, which can reduce the risk of unconsidered factors and improve data quality. VR versions of standard test procedures, e.g., for stress induction, yield comparable effects as real world setups (5–7), and even have improved opportunities for scaling difficulty. Extensive control over task and environmental features also enables multicenter or even out-of-the lab-studies at high quality (8). For these purposes, standalone VR applications can be developed and delivered to cooperating recruitment centers or even directly to the participants.

### Natural interaction and surveillance

Virtual reality allows for natural interactions, because the body and its natural movements can be used as input device just as in the real world. This enables the investigation of natural behavior, which is particularly relevant for eating behavior, which is typically disturbed in situations without external regulation and observation. Further, the motion tracking of the body or body parts that is needed to make them an input device enables new outcome parameters, such as grasping speed (9), for example automatic approach or avoidance tendencies (7), body sway as an indicator for balance performance (10) and embodiment with an avatar (11). Eye-tracking, which was traditionally bound to experimental setups requiring head fixation (12), can be used to enable gaze recording in relatively natural settings (13, 14) and hands-free navigation in setups that need the user to keep still (15). Of note, natural interaction also maximizes user comfort, making surveillance a comfortable side effect of good VR.

### Presence

Presence is a central construct of VR development and refers to the experience of “being there” in a virtual place. In recent years, a consensus has been developed to distinguish presence from immersion (16, 17). According to this definition, immersion refers to the technical implementation of substituting sensory data so that the user gets (dominating) sensory information from the virtual environment and not from the actual environment anymore. Presence, in contrast, refers to the subjective experience of the environment and entails a place illusion (that one is in the virtual environment) and a plausibility illusion (that the experiences are really happening). A high degree of presence is associated with physiological and behavioral realism. That is, users react similar to VR stimuli compared to the real word if presence is high (18). Importantly, presence is not to mistake with an illusion in the clinical sense. Users of VR are permanently aware that they are experiencing a second reality and can still distinguish physical from experienced reality. Hence, VR can be seen as a strong stimulus to shift the attentional focus away from the current situation or as a facilitator to imagination tasks.

### Embodiment

Embodiment is defined as the sense of having a body. While psychological frameworks of body experience traditionally focused on affective and cognitive processing of body representations (19), neuroscientific theories emphasize the formation of body representations through synthesis of multisensory experience (20). The multisensory stimulation in immersive VR enables the experience of embodiment for a virtual body (21), a phenomenon which has been widely studied in terms of technical prerequisites (22–25), assessment of embodiment (21), relation to perception of the actual body (26, 27), identity (28), and effects on motor performance (29, 30) and cognition (31). Virtual embodiment can support the assessment and manipulation of body representation and is increasingly used (32–35). Approaches range from studying specific aspects in body representation (36–38) over conditions for malleability of body representation (22, 39, 40) to virtual exposure paradigms (41). In light of the complex nature of body representation of the actual body, embodiment of virtual bodies and their interaction, current results need to be interpreted with care.

## Virtual reality as a tool for psychotherapy of eating and weight disorders

We distinguish three types of VR applications that are currently piloted for therapy of eating and weight disorders. Figure 1 illustrates how VR distraction and relaxation, (enhanced) virtual exposure, and embodiment experiences draw upon specific properties of VR. Note that the current studies are typically pilot studies; none of the existing tools has so far made its way toward efficacy trials. Nevertheless, we argue that the pilot results can be used for further conceptual and technological development which can potentially transform the tools to helpful add-ons or even alternatives to existing treatments in the future.

Virtual reality relaxation is a broad yet simple intervention that in its current form basically copies an existing approach to VR. VR relaxation has been shown to support acute relaxation and wellbeing in various acute medical settings, including chemotherapy for cancer (42), cardiac surgery (43), labor, and care for veterans with dementia (44) and it also showed high efficacy in reducing acute stress also in psychiatric patients, with a tendency to be even more efficacious than standard relaxation procedures (45). This gives raise to the assumption that the multisensory stimulation through VR may facilitate relaxation for patients who have difficulties to relax. On the conceptual side, further development needs sound assumptions on what typically causes these difficulties. In the case of eating and weight disorders, situation specific aspects such as solitude, time pressure or social stress could play a role, but also difficulties in perceiving stress versus relaxation consciously. Both aspects could be targeted by technological solutions, e.g., by generating personally relevant situations to practice relaxation or by implementing biofeedback to the scenery.

The second big field for therapeutic applications of VR in eating and weight disorders are virtual exposure setups. They were initially developed for the treatment of anxiety disorders, where they yield effect sizes comparable to real-world exposure (46, 47). The main advantage of VR exposure for patients with anxiety disorders is improved availability and flexible adjustment of difficulty. Further, there are hints that patients have fewer reservations about virtual exposure than about real-life exposure (48). For patients with eating and weight disorders, virtual paradigms facilitate exposure to imaginary therapy consequences, such as weight gain or loss, and therefore have the potential to become a valuable tool for increasing motivation for treatment (49–51) and thereby improving therapy adherence.

Exposure to different body weight is currently best piloted in patients with anorexia nervosa. Mölbert et al. (36) demonstrated that VR exposure to weight manipulated versions of their body caused anxiety in patients with anorexia nervosa which faded over the course of the experiment. In preliminary studies, therapeutic exposure to avatars of higher weight showed promising results in addressing fear of weight gain (32, 41, 52). However, detailed mechanisms of this effect, e.g., contributions of physiological habituation versus cognitive restructuring and the most efficient instruction are still unclear and require further investigation (32). Further enhancement with biofeedback (32, 53), multisensory stimulation within VR or attentional direction could be implemented, but also require thorough pilot studies. Since body dissatisfaction and overvaluation of body weight for self-esteem play a role in all eating and weight disorders (54–56), adaptations for obesity and bulimia nervosa should be considered, as well.

Another innovative, yet very preliminary approach for advancing therapy of eating and weight disorders through VR are tools that address body image disturbance through non-exposure procedures involving different degrees of embodiment. According to a recent review by Magrini et al. (32), the new experience of a virtual and the actual body at the same time can trigger at least temporary changes in body representation. For example, Keizer et al. (22) and Serino et al. (39) used a full body illusion of a healthy weight body in a sample of patients with anorexia nervosa and demonstrated that it temporarily modified their experienced body size toward their actual thin body size. Crucially, the exact mechanism and technological and individual prerequisites for this effect are so far not understood. Different pathways of how embodiment influences body image in the short and longer term need to be considered, among them habituation processes and cognitive restructuring (57, 58), but also recalibration in multisensory processing of the body (59). In addition, the Proteus effect, meaning a cognitive bias toward properties of an embodied body, could induce identification with the virtual body and imply more “healthy” cognitions. In order to develop these approaches to meaningful tools for psychotherapy, further research is needed.

## User-centered design as a framework for the development of virtual reality setups

In light of the complexity of virtual environments, but also of symptoms of eating and weight disorders, it is obvious that development of VR tools for clinical research and therapy cannot be successful from the scratch. Rather, iterative piloting with conceptual and technological refinement are essential to produce a good virtual reality tool. Virtual reality setup development is a complex task which requires high levels of multidisciplinary specialized expertise and a structured procedure with long-term perspective. Only recently, user-centered design approaches are established that introduce structured and planned changes to existing setups within feedback loops from applications in research and therapy (60, 61). This approach requires structured interdisciplinary collaboration between clinical staff and specialized computer scientists, and can be facilitated through software sharing under creative commons licenses.

**FIGURE 2:**
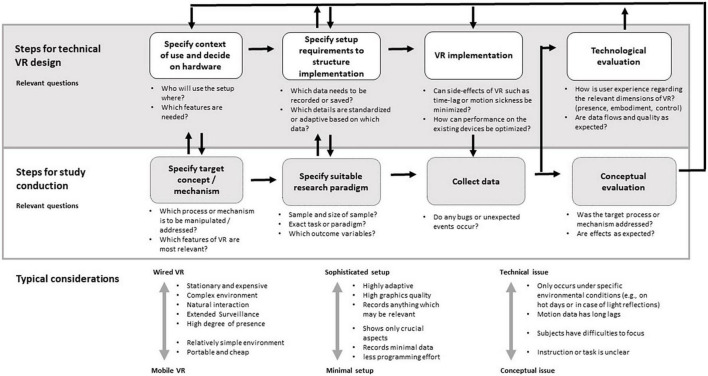
Pipeline for user-centered design of virtual reality (VR) setups for clinical research, helpful questions to discuss for the respective steps and typical considerations.

Figure 2 exemplifies the process of iterative setup development between clinical researchers and computer scientists following a user-centered design approach. Clinical researchers are typically well-trained in necessary steps for conducting studies, but underestimate the need for constant and closely intertwined feedback loops with programmers of their VR environment. User-centered application design is an iterative process that continuously evaluates user requirements and design solutions to these requirements with the aim to optimize the product. Transfer to VR development for clinical purposes includes the following steps to be carried out *additionally* to the usual study conduction process: (1) Analysis of the context in which the setup is to be used and decision for hardware which is appropriate to study aims and context, (2) Specification of exact setup requirements and VR properties that are to be exploited, (3) VR implementation, and (4) Technological evaluation of the setup. The questions and typical considerations provided in Figure 2 can serve as inspiration for discussions between clinical researchers and involved computer scientists, but are certainly not exhaustive.

Virtual reality development forces the designer to produce a relatively rich and complex environment, which implies many design decisions for and against optical and interactive details in the virtual environment. In order to minimize unwanted effects of these decisions and to evaluate the environment on critical VR dimensions, the use of established and standardized questionnaires for evaluation of VR properties [examples are provided in (24, 27, 28, 38)] is essential. Ideally, the technological evaluation of the setup is carried out together with the conceptual evaluation. User ratings of VR experience are not only needed to technically improve the setup, but can also inform the interpretation of the experimental data.

## Conclusion

In this perspective paper, we have summarized the current state-of-the-art in VR technology and research for eating and weight disorders and we have provided a framework within the user-centered design approach that can serve as guideline in the interdisciplinary work and professionalization of setup development. Over the course of the COVID-19 pandemic, telepsychiatry has received a boost in acceptance and dissemination. Two main factors played into the sudden success: A sound empirical basis that telepsychiatry was safe, well-accepted and useful, but also a strong political push that created the organizational prerequisites for broad use (62). Likewise, we consider two aspects as crucial for VR technology to become a valuable component of psychotherapy: It needs a sound empirical basis describing for which problems and patients it is safe, useful and accepted, and its development and maintenance needs professional organization and delivery structures.

As starting point to systematize research on use cases and development of VR for research and therapy of eating and weight disorders, we provided an overview on the specific features of VR and their use in existing approaches for clinical research and therapy of eating and weight disorders. Specifically, we see high potential in setups that use unique features of VR to establish innovative paradigms. Currently, however, most approaches for VR tools get stuck at a very early pilot stage. To encounter this problem, we encourage intradisciplinary efforts involving both clinicians and computer scientists to promote the development of VR tools within long-term, user-centered design processes.

## Data availability statement

The original contributions presented in this study are included in the article/supplementary material, further inquiries can be directed to the corresponding author.

## Author contributions

SB, SS, AK, and KG conceptualized the manuscript. SB drafted the manuscript. All authors critically revised the draft and approved the manuscript.

## Abbreviations

VR: immersive virtual reality technology.
